# Erosion-Corrosion Behavior of 316L Stainless Steel in Chloropropene

**DOI:** 10.3390/ma18010120

**Published:** 2024-12-31

**Authors:** Yingying Dai, Changxin Wan, Shuai Yan, Guandong Wang, Yan Yang, Youwei Liao

**Affiliations:** 1 School of Materials Science and Engineering, Central South University of Forestry and Technology, Changsha 410000, China; 2 School of Physics, Electronics and Intelligent Manufacturing, Huaihua University, Huaihua 418000, China; wanchangxin2010@163.com; 3 Epoxy Resin Factory, Sinopec Hunan Co., Ltd., Yueyang 414000, China; yansh37.hnsh@sinopec.com (S.Y.); wanggd90.hnsh@sinopec.com (G.W.)

**Keywords:** 316L stainless steel, erosion–corrosion, pitting, complex

## Abstract

The objective of this study is to investigate the impact of different pH values and chloropropene flow rates on the erosion–corrosion behavior of 316L stainless steel. The influence of various factors on the surface morphology was analyzed using scanning electron microscopy, X-ray powder diffractometry, and electrochemical impedance spectroscopy techniques. The results revealed that at a pH value of 3.2 and a chloropropene flow rate of 2.20 m/s, soluble transition metal complexes (ferrocene) were observed on the sample surface, which enhanced the erosion–corrosion process by interacting with corrosion products. With an increase in flow velocity and decrease in pH value, the protective resin coating on the sample surface deteriorated, leading to an oxygen concentration difference between exposed and resin-protected areas of the metal substrate. This resulted in localized oxidation–reduction reactions at these sites, forming metal hydroxides (such as FeOOH and Cr(OH)_3_). Therefore, analyzing the effects of flow rate and pH on the erosion–corrosion of 316L stainless steel is of great significance for improving production efficiency, ensuring safety measures, and environmental protection.

## 1. Introduction

Since the commissioning and operation of Unit 100 in a petrochemical organic chlorine facility, the flow of acidic substances such as chloropropene during the production and transportation processes has led to erosion and damage on pipe walls [1,2]. The facility experienced 11 unexpected parking accidents between 2010 and 2021. Among them, 6 cases were caused by equipment corrosion, accounting for 54.55% of the total parking accidents(the frequency of erosion-corrosion is relatively high), as shown in Table 1, resulting in accelerated degradation and reduced service life of equipment and pipelines constructed from 316L stainless steel [3]. Therefore, conducting a systematic analysis and investigation into the mechanism underlying erosion–corrosion in 316L stainless steel holds significant importance for enhancing production efficiency, ensuring safety measures, and environmental protection.

Erosion–corrosion is the outcome of various influencing factors, encompassing erosion angle, medium flow velocity, pH value of the flowing medium, dissolved oxygen concentration, etc. [4,5,6]. When solid particles are present in the flushing fluid, two types of forces act upon the metal surface: horizontal shear force and vertical normal stress. The metal material experiences more severe damage from flushing corrosion at smaller flushing angles of solid particles; however, as the flushing angle increases, the rate of flushing corrosion decreases [5]. Nevertheless, it should be noted that chloropropene investigated in this study is a product from a preceding section intended for use in epichlorohydrin production and does not contain solid particles. The presence of dissolved oxygen aids in forming a passivation film on inert materials, which can mitigate brush corrosion [7]. The section discussed in this article represents a closed pipeline, with minimal variation observed in the dissolved oxygen content. An increase in the flow rate of the medium can lead to enhanced erosion, causing greater damage to the protective film layer on the sample’s surface and consequently increasing the exposed substrate area, thereby accelerating corrosion effects [8]. Different pH values have an impact on the extent of erosion–corrosion damage experienced by metals. Changes in pH induce alterations in both the composition and structure of passive films formed on metal surfaces. When there is a high concentration of H^+^ ions within the erosive medium, resulting in low pH levels, erosion–corrosion processes are accelerated [9].

In recent years, several scholars have conducted research on the impact of flow velocity on the erosion–corrosion rate of stainless steel. For instance, LÓPEZ et al. [10] investigated the erosion–corrosion behavior of two different types of stainless steel under varying velocities. The findings revealed that mechanical erosion predominantly governed the erosion–corrosion process in AISI304 stainless steel, whereas electrochemical corrosion dominated the erosion brush process in AISI420 stainless steel. However, both materials exhibited an increase in surface damage with higher impact velocities. RIHAN et al. [11] demonstrated that at low speeds, fluid-induced mechanical erosion had a relatively limited effect compared to electrochemical corrosion, which controlled the entire process of erosion–corrosion. The erosion–corrosion behavior of Z2CN19·10N stainless steel in liquid–solid two-phase flow was investigated by Wu et al. [12]. Their findings revealed that the erosion–corrosion behavior was significantly influenced by the flow velocity of the Cl-containing solution. Kuz’nicka [13] examined the corrosion of heat exchanger pipes in a water environment with high concentrations of chlorine ions. It was observed that high-speed water flow caused damage to the oxidized copper protective film on the tube surface, allowing fresh electrolytes containing chloride ions to penetrate into the pipeline and leading to subsequent corrosion.

In general, erosion–corrosion is observed to accelerate with increasing fluid flow velocity: mechanical erosion losses also exhibit an upward trend with higher flow velocities, leading to an increased number of metal surface pits that further enhance the chemical corrosion rate. The combined effect of mechanical erosion and electrochemical corrosion synergistically promotes total erosion–corrosion, resulting in a proportional increase in material loss as the flow velocity rises [5,6,14,15]. Previous studies [16] have also substantiated that solutions with higher acidity exhibit a more pronounced erosion–corrosion rate, with the maximum degradation observed at the lowest pH value. However, limited research has been conducted on the influence of pH on the erosion–corrosion of stainless steel.

Previous studies only analyzed the erosion and corrosion caused by the presence of chloropropene water on the 100-unit plant from a theoretical perspective, without conducting detailed data analysis [17]. Based on this, this study explores the relevant performance indicator schemes by taking the actual influencing factors of the factory as the research object. This study focuses solely on investigating the erosion–corrosion behavior of 316L stainless steel by examining various flow rates and pH levels, without accounting for potential confounding factors. The experimental setup involves subjecting the weighed samples of 316L stainless steel to different flow rates (ranging from 0 to 3.30 m/s) and pH values (ranging from 4.2 to 2.8), utilizing a rotating cylindrical electrode. Then, characterize and analyze the samples to study the effects of different factors on the erosion–corrosion of 316L stainless steel. Research the minimum pH range for chloropropene transportation pipelines. Propose an optimization and renovation plan for the 100-unit chloropropene drying system, including flow rate control, pH monitoring, and control measures for chloropropene.

## 2. Materials and Experimental Procedure

### 2.1. Materials and Equipment 

The chemical composition of the 316L stainless steel samples utilized in this study is presented in Table 2, with all sample dimensions measuring 10 × 10 mm. Prior to each experiment, the samples were meticulously polished using sandpaper particles of sizes 400, 800, and 1200 μm consecutively. Subsequently, a polishing agent consisting of diamond paste with a particle size of 3.5 μm was employed. Following the polishing process, the samples were thoroughly cleansed using deionized water and subsequently dehydrated before being dried utilizing anhydrous ethanol. To ensure protection and preservation of the exposed sample surface, epoxy vinyl ester resin was applied as a coating material. After drying, the weight of the film was accurately measured employing an analytical balance with a precision level of up to four decimal places (0.0001 g), while simultaneously recording corresponding data. The main testing and analysis instruments for the experiment are shown in Table 3.

### 2.2. Test Equipment and Process

The electrochemical test was conducted under the following conditions: the erosion–corrosion duration was 100 h, and the test temperature was maintained at room temperature. The flow velocity of liquid transported through pipelines typically ranges between 1 and 3 m/s. This study investigates the flow velocity factors by considering five values (0 m/s, 1.65 m/s, 2.20 m/s, 2.75 m/s, and 3.30 m/s) both above and below this range. The moisture content of chloropropene can influence its pH value. In a petrochemical plant in 2023, the moisture content of chloropropene measured approximately 106.32 ppm (pH = 3.6). To examine the pH factor, this study selected five values (pH = 4.2, pH = 3.9, pH = 3.6, pH = 3.2, and pH = 2.8) above and below this range. The electrochemical test was performed after reaching a stable corrosion potential state. The frequency range for electrochemical impedance measurements ranged from 0.01 Hz to 100 kHz with a disturbance voltage amplitude set at 10 mV. After each experiment concluded, the sample underwent ultrasonic cleaning, and any corrosion products on its surface were rinsed off using deionized water. Subsequently, the sample was air-dried, and data recording included reweighing. Repeating each experiment three to five times ensured reliability.

### 2.3. Characterization

The parameters for X-ray powder diffractometer (XRD) analysis involve employing CuK-α (wavelength 1.5406 Å) as the X-ray source, operating at a voltage of 40 kV and a current of 25 mA, with a step size of 0.02°, a scanning time of 0.5/s, and a scanning range (2θ) from 20° to 80°. Morphology and elemental compositions of corroded samples were investigated using SEM-EDS and X-ray mapping (Phenom Pharos, Thermo Fisher Scientific) at an applied voltage of 15 kV. FTIR testing was conducted within the wavenumber range of 4000~400 cm^−1^ with a resolution set at 4 cm^−1^ and scanned for a total of 32 times.

## 3. Results and Discussion

### 3.1. Corrosion Kinetics

**Figure 1:**
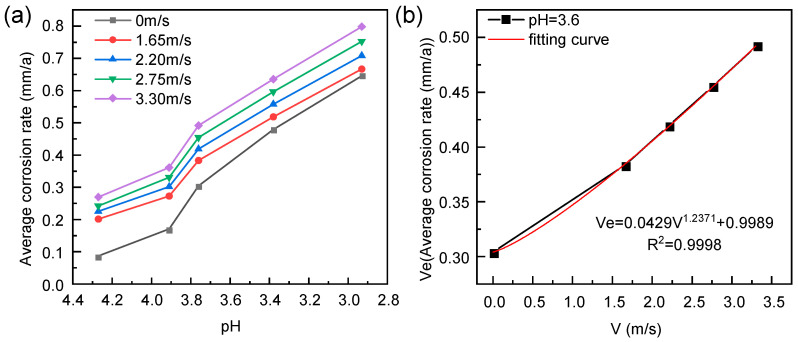
Corrosion kinetics curve of 316L stainless steel under (**a**) the combined influence of current velocity and pH and (**b**) the individual influence of current velocity.

The corrosion rate variations of 316L stainless steel after erosion–corrosion under different pH and flow velocities are depicted in Figure 1a. The corrosion kinetics curve exhibits an overall increasing trend, with the average corrosion rate escalating as the flow velocity rises, thereby promoting halogen ion mass transfer. Erosion disrupts the protective film layer on the substrate, leading to a reduction in its thickness and compromising its integrity, consequently exacerbating corrosion [16]. Moreover, a decrease in pH value results in an increase in the average corrosion rate of the samples. Notably, at a flow velocity of 3.30 m/s, samples exhibit their highest average corrosion rates across various pH values. Specifically, at a pH of 2.8, the sample experiences a maximum corrosion rate approximately equal to 0.7984 mm/a.

The moisture content of a specific petrochemical industrial-grade chloropropene was measured to be 106.32 ppm (pH 3.6). Figure 1b illustrates the average corrosion rate of 316L stainless steel samples subjected to erosion–corrosion processes at various flow velocities, based on the aforementioned parameters. The direct impact of the flowing fluid on the sample surface significantly influences the protection of the substrate’s membrane layer. As the flow velocity of the scouring medium increases, it leads to erosion and compromises the integrity of the resin protective layer on the 316L stainless steel surface. The average corrosion rate of 316L stainless steel exhibits a linear increase with higher flow velocities, indicating that greater damage is inflicted upon the metal material [5]. Without considering erosion angle, the following fitting formula can be employed to describe variations in erosion–corrosion rates with velocity [18]:

(1) $$V_{e}=kV^{n}+C\;V_{e}=0.0429V^{1.2371}+0.99898$$

where *V_e_* and *V* are the average material loss rate and fluid velocity, respectively; the constant *C* is the weight loss at zero fluid velocity; and *K* and *n* are material fitting constants determined by experiments. As shown in Figure 1b, the precision of nonlinear regression analysis using Equation (1) is relatively high, and the coefficient of determination is 0.9999 [19].

### 3.2. Composition Analysis

**Figure 2:**
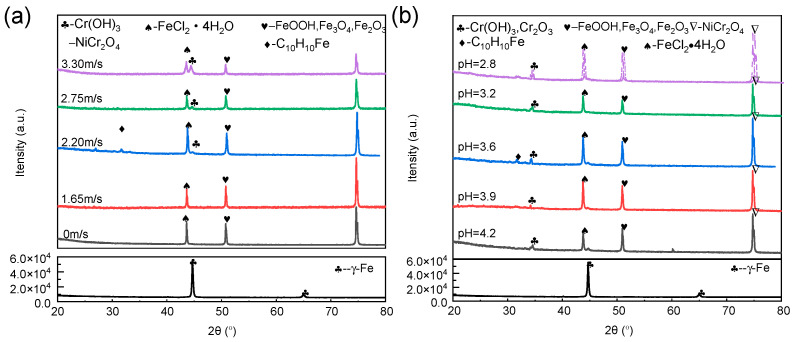
XRD analysis of 316L stainless steel after corrosion at (**a**) different flow rates and (**b**) pH values.

The XRD analysis of 316L stainless steel prior to corrosion is presented in Figure 2, revealing the presence of the γ-Fe phase. Subsequently, the corroded samples were analyzed under constant pH conditions at different flow rates, as depicted in Figure 2a. The predominant corrosion products identified include FeCl_2_·4H_2_O, FeOOH, Fe_3_O_4_, Fe_2_O_3_, NiCr_2_O_4_, and Cr(OH)_3_ during the chloropropene-induced corrosion of 316L stainless steel. These corrosion products accumulated on the surface of stainless steel, forming a product film primarily composed of Fe_3_O_4_ and Fe_2_O_3_ components. It has the characteristics of looseness and weak bonding with the metal matrix. As the flow rate of chloropropene increases, it accelerates the erosion of metals by Cl^−^. Cl^−^ has strong penetration ability, can penetrate the protective layer on the metal surface, reach the surface of the metal substrate, and react with the metal to form soluble compounds, which destroy the density and integrity of the metal surface protective layer, making it easier to peel off from the metal substrate surface, thus accelerating the corrosion of the substrate [15].

The presence of ferrocene (C_l0_H_10_Fe) was detected on the surface of 316L stainless steel at a flow velocity of 2.20 m/s. During the production process of chloropropene, the generation of 1,5-hexane occurs. Due to their characteristic properties as halogenated hydrocarbons and olefins, both chloropropene and 1,5-hexane can form complexes with transition metal ions such as iron, copper, nickel, and olefin [20], resulting in the formation of soluble transition metal compounds like ferrocene. Ferrocene is a homogeneous catalyst. It has a certain degree of reducibility, and the iron in 316L stainless steel is in a relatively active metallic state under the flushing of chloropropene water. When the two come into contact, it is a humid and acidic environment, and ferrocene may corrode iron through redox reactions. It poses challenges in its separation from the product during subsequent processes, thereby resulting in escalated corrosion and other associated complications [21].

The discovery of a corrosion-accelerating complex was made at a flow rate of 2.20 m/s. Consequently, XRD analysis of the corroded samples under different pH conditions, while maintaining a fixed flow rate of 2.20 m/s, is presented in Figure 2b. The phase composition of the corrosion products corresponds to that shown in Figure 2a. Notably, only complexes that promote corrosion were observed at pH 3.6 and a flow rate of 2.20 m/s, indicating the substance’s inherent instability and its propensity to readily react with the metal substrate, thereby accelerating corrosion [21].

**Figure 3:**
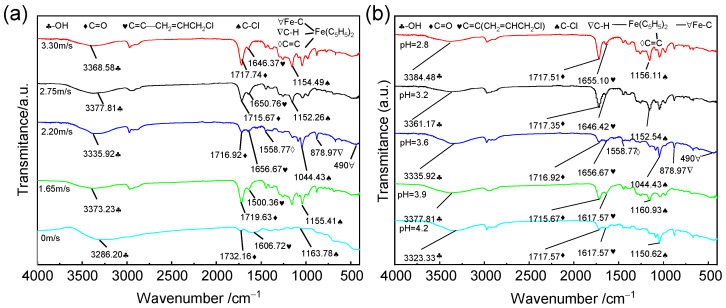
FT-IR analysis of 316L stainless steel after corrosion at (**a**) different flow rates and (**b**) pH values.

The corrosion products were subjected to FT-IR analysis under identical conditions, as depicted in Figure 3. The infrared spectroscopic detection results following erosion–corrosion tests at different speeds and different pH values can be categorized into four distinct regions: the C-Cl bond stretching vibration region of organic halides (wave number: 730–1500 cm^−1^); the -OH bond stretching vibration region of alcohol or phenol bound by hydrogen bonds (wave number: 3400–3200 cm^−1^); the C=O bond vibration region (wave number: 1900–1650 cm^−1^); the C=C double bond stretching vibration region (wave number: 1690–1500 cm^−1^); and the moving area of the C-H out-of-plane bending vibration region (1000–650 cm^−1^) [22]. In addition to these characteristic regions, several additional peaks were observed, including a ferrocene C=C skeleton vibration peak at 1558.77 cm^−1^, a C-H out-of-plane bending vibration peak at 878.97 cm^−1^, and an Fe-C vibration peak at 490 cm^−1^ [23], which is consistent with the XRD product analysis findings illustrated in Figure 2.

As depicted in Figure 3, an increase in erosion speed and a decrease in pH result in enhanced absorption peaks of -OH stretching vibrations. This phenomenon suggests that the flow velocity promotes the forward hydrolysis reaction of chloropropene, thereby facilitating the corrosion rate. In Figure 3a, it can be observed that the absorption peaks of C-Cl at wave numbers 1163.78, 1155.41, 1044.43, 1152.26, and 1154.49 cm^−1^ initially exhibit strength followed by weakening trends, with the highest absorption peak occurring at a flow rate of 2.20 m/s. Although generated ferrocene exhibits certain anti-corrosion properties, it does not form strong surface bonds with the sample material, rendering it ineffective as an anti-corrosion agent. Subsequently, the absorption peak weakens further, and corrosion intensifies over time. In Figure 3b, a decrease in pH value is observed to weaken the absorption peak of C-Cl, indicating increased corrosiveness. Figure 3 demonstrates accelerated corrosion only at a pH level of 3.6 and a flow rate of 2.20 m/s. This indicates that the substance is inherently unstable and prone to react with metal substrates, leading to accelerated corrosion [21].

### 3.3. Morphology Analysis

**Figure 4:**
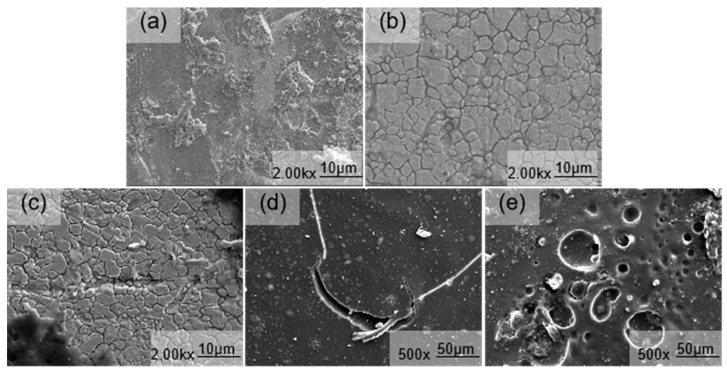
SEM images of the surface morphology of 316L stainless steel subjected to erosion–corrosion processes at different flow velocities ((**a**) 0 m/s, (**b**) 1.65 m/s, (**c**) 2.20 m/s, (**d**) 2.75 m/s, and (**e**) 3.30 m/s).

The SEM micrographs in Figure 4 depict the erosion-induced surface morphology of 316L stainless steel specimens at various flow velocities under constant pH conditions. It is observed that the characteristics of corrosion defects, specifically circular pits, undergo changes with increasing flow velocity during erosion–corrosion processes. Additionally, the surface morphology of the sample exhibits two notable characteristics, i.e., irregular impressions and pits, as well as corrosion pits resembling those observed in previous studies [5]. The mass transfer primarily occurred through natural convection when the surface membrane of 316L stainless steel remained intact, as depicted in Figure 4a. This phenomenon took place under relatively stationary conditions without induced convection, while the passivation film on the metal surface effectively shielded it from severe electrochemical corrosion. Metal passivation played a dominant role, with erosion wear being comparatively weak. Throughout the entire erosion–corrosion process, electrochemical corrosion emerged as the predominant mechanism. As illustrated in Figure 4b, slight detachment of the surface coatings resulted in darker indentations and exposed fresh metal surfaces. Figure 4c,d demonstrates an uneven corrosion product film and peeling phenomena on its surface layer. The erosion trajectory exhibited irregularities characterized by numerous scratches. Severe damage to both the surface passivation film layer and resin led to deeper grooves aligned parallelly. This occurrence likely stemmed from fragments peeling off from the substrate that facilitated flow velocity-induced erosion.

Additionally, the induced convection enhances mass transfer, while the shear force generated by liquid flow disrupts the passivation film on the metal substrate surface, leading to a rapid increase in corrosion rate. At this stage, erosion and wear become dominant factors. As depicted in Figure 4e, pitting occurs on the material’s surface. The elevated flow velocity intensifies corrosive media transmission, thereby promoting contact between the substrate and Cl^−^. Due to Cl^−^’s strong penetration ability, it damages the oxide film on the material’s surface and induces pitting. These pits propagate longitudinally, posing a risk of corrosion perforation. Moreover, defects like pitting are often accompanied by negative potential, making them preferentially corroded as anodes while creating cathodic protection around them [5]. Therefore, erosion–corrosion is frequently associated with galvanic corrosion. Simultaneously, as flow velocity increases, material surface roughness also rises correspondingly, which augments specific surface area formation of galvanic cells and accelerates corrosion rates. Furthermore, with increasing speed, the mass transfer coefficient also increases [6]. Starting from Figure 4c, ferrocene presence leads to accelerated corrosion, causing intensified degrees of corrosion.

**Figure 5:**
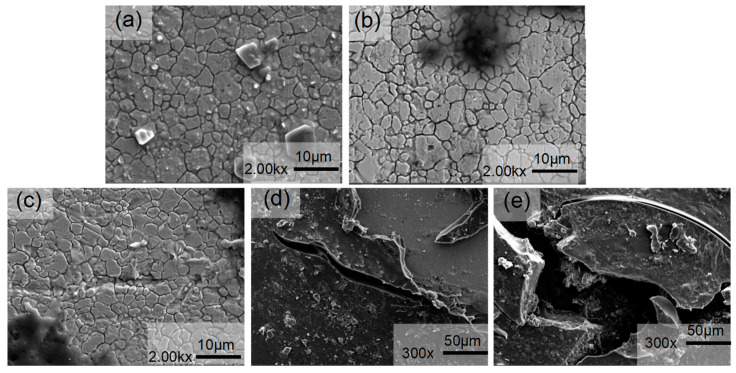
SEM images of the surface morphology of 316L stainless steel subjected to erosion–corrosion processes at different pH values ((**a**) 4.2, (**b**) 3.9, (**c**) 3.6, (**d**) 3.2, and (**e**) 2.8).

The SEM images in Figure 5 depict the erosion–corrosion effects on 316L stainless steel at various pH values under a flow velocity of 2.20 m/s. The characteristics of corrosion defects exhibited variations with decreasing pH levels. In Figure 5a, the resin layer present on the surface of the 316L stainless steel specimen was effectively removed by the medium, resulting in the destruction of the oxide film and exposure of the substrate underneath. Consequently, this liquid phase medium exerted a corrosive influence on the substrate, leading to a reduction in corrosion performance. Furthermore, as pH decreased, corrosion intensified, causing localized detachment of corrosion products and exposing fresh substrate.

The corrosion product film in Figure 5b exhibited localized cracking and the formation of small pits, resulting in anodic dissolution of the metal within the pits and a cathodic reduction reaction outside. This process led to an increased concentration of metal cations within the pores. To maintain electrical neutrality, Cl^−^ ions from outside the pore migrated through the corrosion scale and reacted with metal ions inside, forming metal chlorides. Hydrolysis of these chlorides further reduced pH levels within the pores, accelerating metal dissolution. Eventually, dimple-like pitting corrosion formed on the metal surface as depicted in Figure 5c. With continued decrease in pH (Figure 5d), both the number and size of pitting pits increased, expanding into elongated pits with a small tail appearing on one side. The initial pit formation acted as a flow disturbance, inducing microturbulence around the anode area. This micro-turbulence propagated along with flow direction at a similar opening angle as “flow marks”, dissipating its energy over distance away from the initial pit location. Local permeability decreased with increasing distance from the initial pit, leading to narrow tail formation at the end of the “flow mark”. Vertical evolution of pitting pits posed a risk for corrosion perforation in 316L stainless steel (Figure 5e) [14]. In summary, as pH decreased, corrosion severity intensified, resulting in a gradual increase in the radius and depth of surface-generated pits. Increased corrosiveness due to the solution’s enhanced corrosive performance (decreased pH) was accompanied by the appearance of solid particle plowing marks on the surface along with certain corrosion products facilitating the erosion of 316L stainless steel [15].

**Figure 6:**
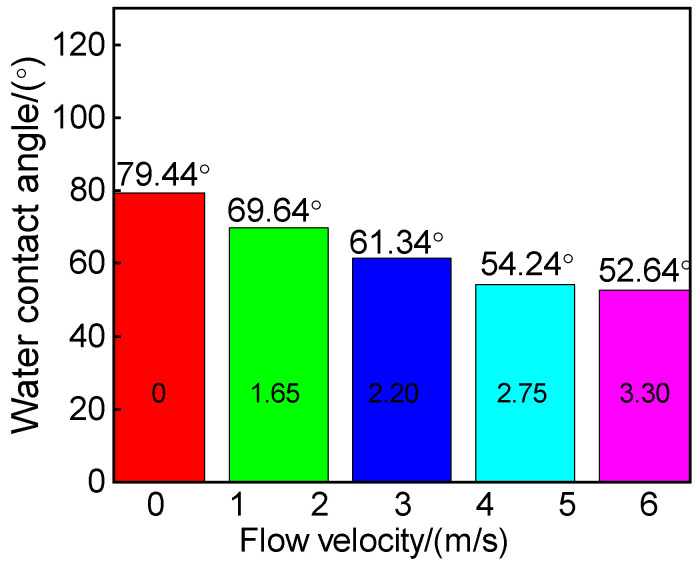
Surface water contact angle of 316L stainless steel after corrosion at different flow velocities.

The water contact angle test results of 316L stainless steel samples after undergoing erosion–corrosion at different flow velocities are presented in Figure 6. It is observed that the sample exhibits the highest water contact angle at a flow velocity of 0 m/s. However, as the flow velocity increases, there is a significant decrease in the water contact angle from 79.44° to 52.64° for the test piece. This phenomenon suggests an inverse relationship between surface roughness and contact angle, indicating that the corrosion product film formed on the metal surface after erosion and corrosion possesses high surface roughness. Consequently, an increased number of micropores on the material’s surface leads to a larger effective surface area and enhanced interaction force between liquid and material surfaces when in contact with each other. This enhanced interaction facilitates liquid penetration into the interior of the material, resulting in reduced contact angles and the formation of a more hydrophilic surface layer [14]. These findings align with SEM analysis results depicted in Figure 4, where it can be observed that increasing erosion speed leads to an increase in surface pores.

**Figure 7:**
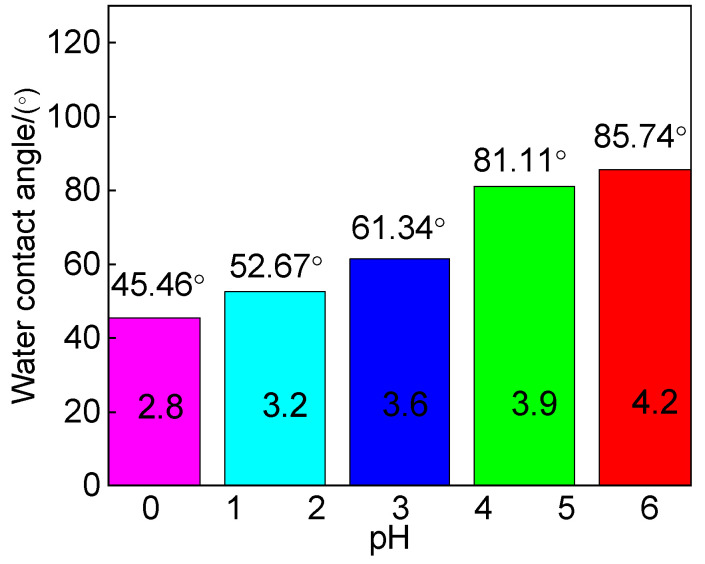
Water contact angle of 316L stainless steel after erosion and corrosion tests at different pHs.

The contact angle of the reactive material exceeds 90° for drainability and is below 90° for waterproofness. Figure 7 illustrates the results of water contact angle tests conducted on 316L stainless steel samples at various pH values following erosion–corrosion. The water contact angle of the stainless steel was found to be less than 90°, indicating a hydrophilic nature with low surface energy and a tendency to easily become wet by water. Notably, the highest water contact angle was observed at a pH value of 4.2. With an increase in pH, there was a significant rise in the water contact angle from 45.46° to 85.74°, suggesting considerable changes in surface roughness due to metal oxide formation during erosion–corrosion reactions on stainless steel surfaces. This roughened surface exhibited poor waterproofness as greater surface roughness led to smaller contact angles by creating more small holes on the material’s surface and consequently increasing its effective area for liquid penetration into it, resulting in a more hydrophilic surface layer [14]. These findings align with those obtained from SEM analysis presented in Figure 5.

### 3.4. Electrochemical Impedance Spectroscopy

**Figure 8:**
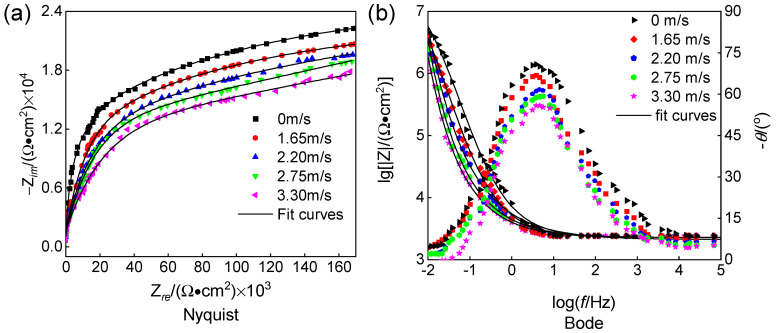
Nyquist curves and their corresponding Bode spectra of 316L stainless steel at different flow velocities ((**a**) Nyquist and (**b**) Bode).

The Nyquist and Bode plots of 316L stainless steel specimens under erosion–corrosion at different flow velocities are presented in Figure 8. The Nyquist plot (Figure 8a) reveals that the capacitance arc radius corresponding to high flow velocity is significantly smaller compared to that associated with low flow velocity. Simultaneously, the Bode plot (Figure 8b) demonstrates a larger phase angle for low flow velocity. A greater capacitance arc and phase angle often indicate the presence of a more effective protective passivation film. Notably, as the flow velocity increases, the diameter of the capacitive arc decreases, suggesting reduced corrosion resistance. This phenomenon aligns with the average corrosion rate depicted in Figure 1b.

**Figure 9:**
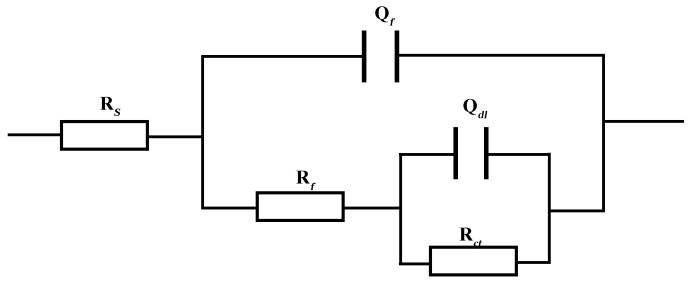
Equivalent circuit.

The impedance data were fitted using the selected equivalent circuit model shown in Figure 9. In Figure 9, R*_s_* represents the solution resistance; R*_ct_* denotes the charge transfer resistance; R*_f_* signifies the resistance of the generated corrosion product film; Q*_dl_* represents the interface double layer capacitance; Q*_f_* indicates the film layer capacitance; and R*_p_* corresponds to the polarization resistance throughout the entire reaction process. The fitted data can be found in Table 4. In passivation systems, R*_p_* is typically inversely proportional to the passivation current density and serves as an indicator of protective properties exhibited by the passivation film. R*_p_* = R*_f_* + R*_ct_*; a smaller value of R*_p_* implies poorer protection provided by the passivation film and lower corrosion resistance of the passivation material [24].

**Figure 10:**
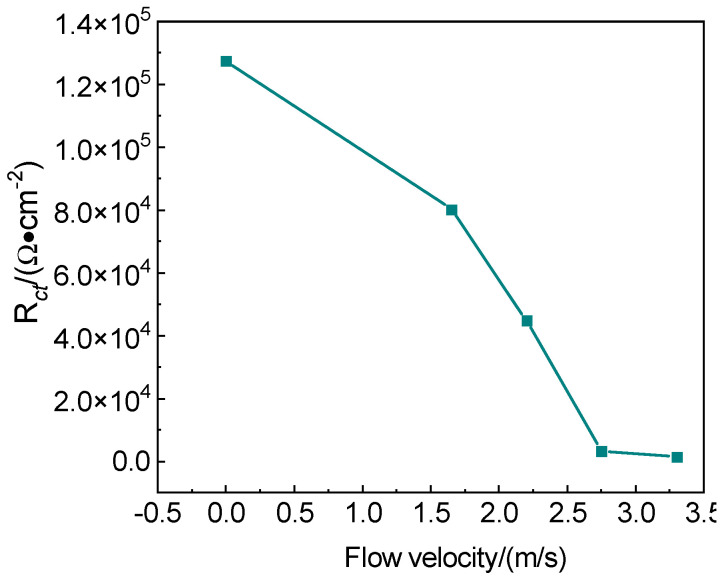
Variation of charge transfer resistance with flow velocity.

Figure 10 shows the trend of charge transfer resistance as a function of flow velocity. The charge transfer resistance (R*_ct_*) of the specimen under erosion–corrosion conditions decreases with increasing flow velocity, and the charge transfer resistance reflects the susceptibility of the specimen to corrosion. Under the erosion of chloropropene at a flow velocity below 2.5 m/s, R*_ct_* rapidly decreased, possibly due to the detachment of corrosion products formed on the surface of 316L stainless steel under the erosion of flowing media. Under the erosion of chloropropene at a flow velocity exceeding 2.5 m/s, a thinner passivation film and loose corrosion products gradually formed on the surface of 316L stainless steel. Then, the film eventually ruptured and fell off, decreasing R*_ct_*. The larger the Rct, the lower the corrosion rate of the sample at low flow velocities. This phenomenon occurred because as the flow velocity of the medium increased, the solubility of oxygen in the flowing medium decreased. Although the corrosion product film is easier to form, the diffusion rate of oxygen in the flowing medium increases. Consequently, the diffusion of O_2_ and Cl^−^ leads to an increase in charge transfer rate. As a result, the conductivity of the medium increases, leading to an increase in the porosity of the film surface [7,22]. The corrosion product film is not dense, leading to an accelerated corrosion rate.

**Figure 11:**
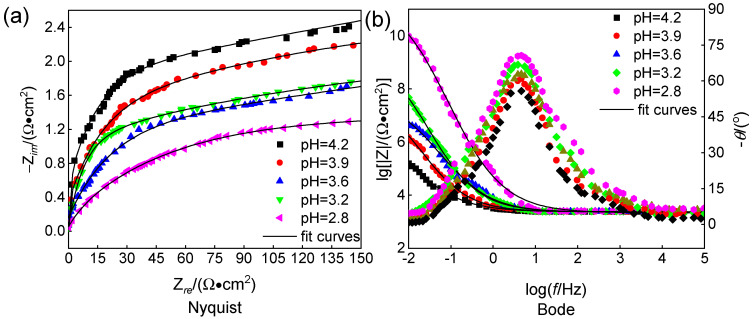
Nyquist curves and their corresponding Bode spectra of 316L stainless steel at different pH ((**a**) Nyquist and (**b**) Bode).

The Nyquist and Bode plots of 316L stainless steel, tested at a flow speed of 2.20 m/s and different pH values under erosion–corrosion conditions, are presented in Figure 11. As depicted in Figure 11a, the diameter of the capacitive area increases with decreasing pH, indicating reduced corrosion resistance and an elevated corrosive rate. In Figure 11b, it can be observed that as the pH decreases, the sample’s resistance continues to decrease while the frequency characteristics curve narrows, and its spectral characteristics decline. This leads to an increase in the erosion–corrosion rate and gradual degradation of the surface membrane on the 316L stainless steel. The increase in water content of chloropropene ionizes more Cl^−^, leading to a decrease in pH. Due to their small radius, chloride ions possess a strong penetration force, which effectively infiltrates gaps within oxide films and reaches metal surfaces, leading to the formation of soluble compounds. Consequently, this process damages the density and integrity of metal surface oxide membranes, ultimately causing substrate corrosion [5,6]. These findings align with those obtained from erosion–corrosion behavior analysis shown in Figure 1. 

The equivalent circuit model employed is identical to that illustrated in Figure 9; fitted data are provided in Table 5, which demonstrates a gradual reduction in R*_p_* accompanied by increased corrosiveness with decreasing pH [25].

### 3.5. Erosion–Corrosion Mechanism

Chloropropene has the characteristics of both olefins and halogenated hydrocarbons [24]. In addition, the chlorine atom in the molecule is very active. It can easily undergo many reactions, such as pro-nuclear replacement and elimination, which are similar to chloroalkanes. Plant-based chloropropene often contains moisture. It produces HCl in a replacement reaction with water as follows:

(2) $${CH}_{2}=CH{CH}_{2}Cl+H_{2}O⇌{CH}_{2}CH-{CH}_{2}OH+HCl$$

The above reaction is reversible in the absence of NaOH, and the resulting HCl undergoes ionization into H^+^ and Cl^−^ when dissolved in water. The presence of ionized H^+^ leads to a decrease in pH, particularly in chloropropene, which already exhibits acidic properties. In this acidic environment, metals are susceptible to electrochemical corrosion, resulting in the release of hydrogen gas. Due to the smaller radius of Cl^−^, it can easily penetrate the passivation film and adhere to the metal surface. Moreover, it displaces oxygen within the passivation film, leading to the formation of highly corrosive chlorides that have detrimental effects on stainless steel [25,26,27].

**Figure 12:**
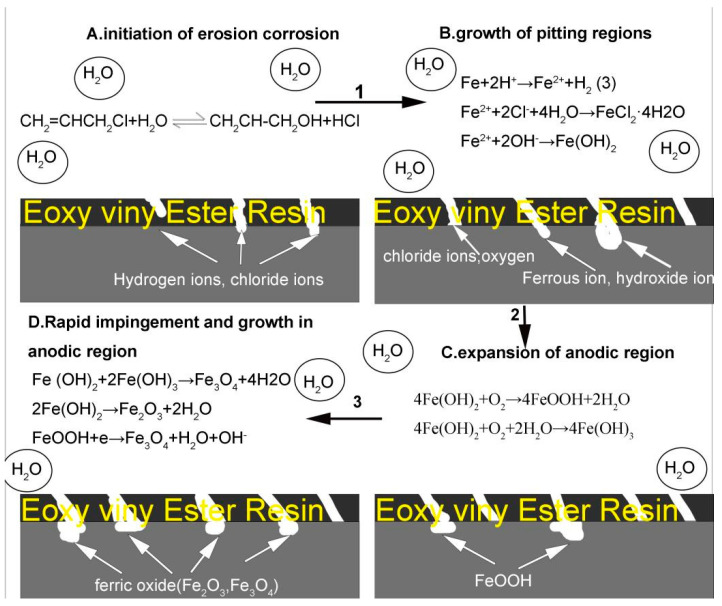
Erosion–corrosion for 316L stainless steel in chloropropene.

The presence of Cl^−^ ions induces a weakly acidic surface medium on stainless steel. Moreover, the corrosion mechanism is illustrated in Figure 12. Chlorine ions compete with oxygen for adsorption sites, resulting in partial oxygen depletion from the stainless steel surface. This hinders the formation of a dense corrosion product layer on 316L stainless steel and promotes pitting corrosion. With increasing flow velocity and decreasing pH, Cl^−^ reacts with iron to form metal chlorides, leading to pore formation within the corrosion product layer. These pores compromise the integrity of the corrosion product layer on 316L stainless steel, facilitating diffusion of Cl^−^ ions back into this layer through these openings. Subsequently, at the interface between the metal matrix and the corroded product layer, chloride ions react again with this layer, causing reduced adhesion between them and eventual delamination of the corrosion product layer. Consequently, susceptibility to corrosion is enhanced in the stainless steel substrate [28].

Different flow velocities and pH values can lead to distinct surface microstructure states. At relatively low flow velocities (high pH), the impact-induced surface damage is relatively weak, with no significant signs of damage observed, and the oxide film exhibits a tight density [16]. With an increase in flow velocity, pitting occurs on the surface of 316L stainless steel, resulting in a decrease in oxide film density. The increased fluid impact energy prevents timely repair through the repassivation process, leading to the destruction of the oxide film. When the flow velocity reaches an optimal level, the shear force exerted by the fluid medium increases. Consequently, a thin corrosion product film forms, exposing fresh metal substrate. As the experiment progresses, this process continues to promote corrosion occurrence. Eventually, sheet-like scratches caused by cutting and dimples caused by vertical impact are observed on the metal surface [14,15].

## 4. Conclusions

A series of experiments were conducted to investigate the erosion–corrosion behavior of 316L stainless steel under different pH values (ranging from 4.2 to 2.8) or chloropropene flow rates (ranging from 0 to 3.30 m/s). The conclusion can be summarized as follows:

1. The erosion–corrosion rate and surface roughness of the sample increased with higher flow velocity and lower pH. At a constant pH of 3.6, SEM analysis revealed that at flow velocities below 2.75 m/s, corrosion was primarily controlled by electrochemical processes, resulting in irregular indentations and pits on the surface. 

2. At a constant pH of 3.6: When the flow rate exceeds 2.75 m/s, both electrochemical corrosion and erosion wear contributed to dimple-shaped corrosion pits. As the flow velocity increased further, iron and chromium oxides were predominantly formed as corrosion products on the sample’s surface. However, at a flow velocity of 2.20 m/s, soluble transition metal complexes (ferrocene) were generated on the surface, forming stable complexes with corrosion products and leading to intensified corrosion. To effectively mitigate corrosion, it is recommended to maintain a medium flow velocity around 2.20 m/s without exceeding 2.75 m/s.

For example, the flow velocity of the medium can be reduced by reducing the diameter of the pipeline and increasing the length of the pipeline. Liquid flow meters can also be added to the device to calculate velocity based on liquid flow rate and pipeline cross-sectional area. Adding pipeline pumps and outlet valves to the equipment to regulate the flow rate has achieved the goal of controlling the flow rate.

3. At a constant flow rate of 2.20, with the increase in pH, the microstructure of 316L stainless steel surface presents corrosion pits with irregular indentations, pits, and dimples. Vertical development of pitting pits poses a risk of corrosion perforation.

4. Additionally, increasing flow velocity coupled with decreasing pH value resulted in the destruction of the protective resin layer on the surface, which created an oxygen concentration difference cell between exposed metal substrate areas and resin-protected ones. The bare metal substrate acted as an anode while the resin-protected metal substrate served as a cathode undergoing oxidation–reduction reactions leading to the formation of hydroxides such as FeOOH and Cr(OH)_3_.

The main causes of erosion–corrosion of 316L stainless steel in chemical environments are the presence of organic chlorides and water. Organic chlorides hydrolyze to form HCl, which dissolves in water and ionizes corrosive chloride ions. The chloride ion radius is small, and the chloride ion content exceeds the critical value of stainless steel resistance to chloride ion corrosion, which will damage the dense internal rust layer and promote further dissolution of the stainless steel substrate. This article focuses on the influence of flow rate and pH on the erosion–corrosion of 316L stainless steel, without considering the influence of the erosion angle of the medium at the corner of the pipeline on the erosion–corrosion of 316L stainless steel, as well as the influence of dissolved oxygen content at the unsealed facility of the organic chlorine 100 units on the erosion–corrosion of 316L stainless steel. In the future, the impact of these two influencing factors or other influencing factors on the erosion–corrosion of 316L stainless steel should be further explored based on the on-site erosion–corrosion situation.

## Figures and Tables

**Table 1:** Summary of unplanned shutdown of organic chlorine unit in Baling Petrochemical [3].

	Reasons for Work Stoppage	Number of Shutdowns	Proportion	Corrosion Proportion
1	System carbon cleaning	3	27.27	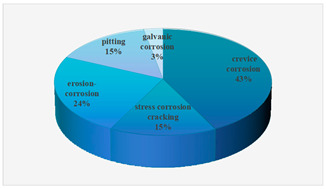 Summary of internal corrosion failure events
2	Electrical instrument fault	1	9.09	
3	Equipment corrosion	6	54.55	
4	Equilibrium production	1	9.09	

**Table 2:** Composition of 316L stainless steel.

Element	C	Si	Mn	P	S	Cr	Ni	Mo	Fe
Content/wt%	0.014	0.520	1.150	0.028	0.003	17.200	13.400	2.400	64.800

**Table 3:** Main experimental apparatuses.

Name	Specification	Manufacturer	City, and Country
Analytical balance	FA2004	Shanghai Optical Instrument Factory No. 1	Shanghai, China
magnetic stirrer	DF-101S	Changzhou Maikono Instrument Co., Ltd.	Changzhou, China
Automatic film applicator	BGD-519	Guangzhou Biaogeda Laboratory Instrument Supplies Co., Ltd.	Guangzhou, China
X-ray diffraction	Ultima IV	Nippon Science&Technology Co., Ltd.	Tokyo, Japan
Fourier transform infrared spectroscope	Is-50	Thermo Fisher Scientific	Waltham, MA, USA
Scanning electron microscope.	TESCAN MIRA4 LMH eds: One Max	Czech Taisken Co., Ltd. (Shanghai)	Shanghai, China
Electrochemical workstation	Reference 600	Gamry	Philadelphia, PA, USA
Contact angle machine	OCA-1 Instruments	DataPhysics GmbH	Stuttgart, Germany

**Table 4:** Electrochemical corrosion parameters of 316L at different flow velocities.

Flow Velocities/(m/s)	R*f* /(Ω·cm^2^)	Error (%)	R*ct* /(Ω·cm^2^)	Error (%)	R*p* /(Ω·cm^2^)	Error (%)	R*_s_* /(Ω·cm^2^)	Error (%)	Q*_f_*		Q*_dl_*	
									Y_0_ /(Ω^−1.^cm^−2^·s^n^)	*n*	Y_0_ /(Ω^−1.^cm^−2^·s^n^)	*n*
0	1.33 × 10^4^	7.32	12.75 × 10^4^	8.24	14.08 × 10^4^	6.72	1.09 × 10^4^	6.23	2.86 × 10^−^^4^	0.87	8.63 × 10^−^^4^	0.83
1.65	4.47 × 10^4^	4.56	8.01 × 10^4^	3.66	12.48 × 10^4^	2.43	8.59 × 10^3^	3.77	7.24 × 10^−^^5^	0.91	2.66 × 10^−^^3^	0.82
2.20	7.02 × 10^4^	7.99	4.49 × 10^4^	8.31	11.51 × 10^4^	5.44	6.01 × 10^3^	6.44	3.34 × 10^−^^5^	0.71	7.03 × 10^−^^3^	0.82
2.75	10.59 × 10^4^	6.23	0.32 × 10^4^	5.23	10.91 × 10^4^	4.88	1.73 × 10^3^	6.01	2.18 × 10^−^^5^	0.85	9.44 × 10^−^^3^	0.80
3.30	10.62 × 10^4^	4.14	0.14 × 10^4^	4.99	10.76 × 10^4^	4.20	0.02 × 10^3^	3.32	2.06 × 10^−^^5^	0.77	13.50 × 10^−^^3^	0.82

**Table 5:** Electrochemical corrosion parameters of 316L in different pHs.

pH	R*f* /(Ω·cm^2^)	Error (%)	R*ct* /(Ω·cm^2^)	Error (%)	R*p* /(Ω·cm^2^)	Error (%)	R*_s_* /(Ω·cm^2^)	Error (%)	Q*_f_*		Q*_dl_*	
									Y_0_/(Ω^−1.^cm^−2^·s^n^)	*n*	Y_0_/(Ω^−1.^cm^−2^·s^n^)	*n*
4.2	5.44 × 10^4^	6.92	7.02 × 10^4^	6.66	12.46 × 10^4^	7.22	7.86 × 10^3^	6.83	6.36 × 10^−^^5^	0.90	4.32 × 10^−^^3^	0.83
3.9	6.02 × 10^4^	5.06	6.32 × 10^4^	4.38	12.34 × 10^4^	5.17	7.23 × 10^3^	4.56	5.91 × 10^−^^5^	0.74	5.96 × 10^−^^3^	0.80
3.6	7.02 × 10^4^	7.12	4.49 × 10^4^	7.99	11.51 × 10^4^	6.82	6.01 × 10^3^	6.43	3.34 × 10^−^^5^	0.71	7.03 × 10^−^^3^	0.82
3.2	9.30 × 10^4^	5.41	2.05 × 10^4^	6.06	11.35 × 10^4^	5.41	4.02 × 10^3^	5.34	3.18 × 10^−^^5^	0.88	8.31 × 10^−^^3^	0.79
2.8	10.3 × 10^4^	7.23	0.92 × 10^4^	6.29	11.22 × 10^4^	6.14	2.02 × 10^3^	7.14	3.23 × 10^−^^5^	0.82	8.36 × 10^−^^3^	0.84

## Data Availability

Data is contained within the article.
